# Building communities of trust: Challenges for disability

**DOI:** 10.4102/ajod.v3i2.77

**Published:** 2014-06-04

**Authors:** Alexander M. Phiri

**Affiliations:** 1 CEO, Southern African Federation of the Disabled (SAFOD), Bulawayo, Zimbabwe

## Abstract

This article asks questions about power and partnership in disability research in Africa. Research has been located too much in one type of organisation or another and not sufficiently in the interaction between a range of legitimate stakeholders. Across Africa and Europe, and government and civil society dialogues, the African development research agenda must be owned by Africans. Fully inclusive national and international research partnerships are crucial, but they must be driven from Africa. European constructions of and interventions concerning people with disability have often been inhumane, seeking to eliminate them from society. African cultures have also stigmatised people with disability. I call for a new African-driven research agenda that promotes the human rights of people with disability, and has people with disability not only participating in this research, but directing it. The Southern African Federation of the Disabled (SAFOD) Research Programme (SRP) is breaking new ground in this regard by allowing ‘the researched’ to become ‘the researcher’.

## The context for this posthumous contribution

In November 2011, the third African Network for Evidence-to-Action on Disability (AfriNEAD) symposium, with the theme ‘building communities of trust’, was held in Zimbabwe. This AfriNEAD symposium was hosted by the Southern African Federation of the Disabled (SAFOD). Mr Alexander Mwanza Phiri, the CEO of SAFOD, was a critical role player in the preparations for hosting this symposium. He died in May 2011, however. We share this article as his legacy and an attempt to continue the dialogue of building communities of trust. All of the ideas in this article were expressed by Mr Phiri in his personal capacity and should not necessarily be taken to reflect the views of SAFOD, past or present. This article has been revised and edited to make it suitable for this special issue by Prof. Mac MacLachlan (Trinity College Dublin) and Dr Gubela Mji (Stellenbosch University).

## Questioning research on disability and development

I want to consider the importance of research and its impact on the policy development agenda in Africa. I also, at the outset, want to recognise that this raises some important and perhaps uncomfortable questions. Who is and who should be driving the research agenda in Africa? There are developed countries and developing countries; and the issue of race, class, tribes, minorities and the majority – who is leading the process? What about government and civil society – are they of any influence in setting the research agenda? Do they work together or in separate ways? What about different sectors of civil society, the non-governmental organisation (NGO) sector, the private sector, and institutions of higher learning, et cetera; to what extent do they embrace each other when lobbying for relevant and appropriate laws governing human development? What about people with disability and people without – do we need each other? What caused the rise of Disabled People’s Organisations (DPOs) when they walked out of a Rehabilitation International conference in Winnipeg in the early 1980s? People without disability may ask: ‘Do we need people with disability to do research on disability?’ People with disability may equally ask: ‘Do we need people without disability to do research on disability?’ And in all of this, just what is the role of civil society?

## Researching as an interdependence entity

I can go on and on asking questions, which is, in fact, what most researchers spend their time doing, often writing volumes of text in the process but finding very few, and sometimes no useful, answers to their questions. I am not saying that people, or researchers for that matter, should not ask questions and try to find answers to these questions. Researchers need to set questions and indeed work on the solutions; but as they do so, I believe they need to reach out and work together. We need to respect one another’s environment; we need to respect each other’s situation and position. There may be distinctions in terms of who we are, where we live or where we come from; what position we hold in society; whether we are in or outside government; whether we are black or white; have a disability or not – we need to find a way of working together because the essence of life is that every human being is important. Each institution is important in its own way; hence the need for all of us to work together in our pursuit of the research agenda. We need to support one another.

## Owning the African research agenda

Unfortunately, the distinction between developed and developing societies, for example, is often that of one group of researchers or academics dominating the other (MacLachlan, Carr & McAuliffe 2010). I hate racism because it should not have any space in this modern world; but the issue of race is critical in the research agenda in Africa and there is a need to address this issue. Africa faces the greatest challenge of establishing and making use of its own research for effective decision-making in development programmes and policy-making. However, our budgets for research and development are not sufficient and can be augmented by those of our colleagues in developed countries. Also, our own governments are not as committed as those in developed countries claim to be to evidence-based actions. We therefore require research staff from developed countries to support the development of the research evidence which is so critical for effective policy development and implementation in our countries.

Our research budgets in Africa should be increased so that we generate our own data to back up our campaigns for meaningful development. We Africans have for too long relied on external researchers, on externally generated data and externally driven research agendas that do not effectively address our issues. If we do not own the research how can we address our needs? African people must invest in home-grown research capacity and research solutions that will meet the specific needs of Africa. This, however, does not mean that we are saying ‘no’ to international partnerships. Yes, we want to work with our international partners, but they should allow us to drive the research agenda. Our partnership should be genuine and based on the principle of equality.

Dr Sindiso Ngwenya (2009), Secretary General of the Common Market for Eastern and Southern Africa (COMESA), maintains that there is a need for Africans to collect their own data to tell the African story, and to benchmark themselves against development targets. Ngwenya actually warns that if we do not collect our own data we will make plans using the wrong data. I agree with him. We need to promote a kind of research which is useful; research that solves daily problems and makes a positive impact on people’s lives, rather than research that is merely for academic purposes. We should not look only at researchers in universities in developed countries as the traditional research community, but also at the emerging research tools and initiatives in developing countries that are embedded in the rich, strong African culture, as being equally authentic for the work at hand.

## Challenging European agendas

Research on disability stemming from developed countries has developed a range of foci, some of which I am quite unhappy about as a person with a disability. For example, the forced sterilisation that girls with mental disabilities are sometimes subjected to is not an African practice but a European one. Another example is the growing practice of encouraging pregnant women to terminate their pregnancies when it is found that the baby they are carrying has a disability (Mitchell & Snyder 2003). I am of the strong opinion that abortion, which has found its way in many African constitutions, is foreign to Africa and should be rejected by our policy makers – more so when it denies innocent children with disability the opportunity to live. Abortion, which has its roots in Europe, is un-African.

Recent history tells of the campaign in Europe to create and preserve a ‘master race’; a special type of people who needed to have the right eye colour, the correct height and so on. We all know what happened: that scientifically and politically driven campaign resulted in one of the world’s most tragic episodes of mass murder of men, women and children – many of them because of their disability.

Euthanasia is another campaign that appears to have thinking in common with forced sterilisation and abortion as ‘acceptable’ methods of reducing the population of people with disability. However, euthanasia is widely talked about and even practiced in Europe and other developed countries. It has yet to take root in Africa – and we have to stop it!

One common feature of these anti-life practices is that they are targeting the elimination of people with disability as part of a solution to ‘the problem of’ disability. This is a sad development for both the disability movement and Africa which, by the way, also has traditions of discriminating against people with disability through oppressive and stigmatising cultural and tribal beliefs (Ingstad & Whyte 1995). In many parts of Africa people with disability were seen, and in many respects continue to be seen, as an abomination, as sub-standard human beings who are a result of sorcery and witchcraft.

## Disability rights and research participation

Africa still needs to make amends in promoting the rights of people with disability as full citizens who are entitled to all human rights. Fortunately, through the rise and work of DPOs there are strong indications and cases of communities that are beginning to embrace people with disability as human beings. For example, there are women and couples who refuse to accept prescribed abortion programmes by carrying pregnancies to full term even after being told of the disability of their unborn child, and who celebrate after giving birth to a child with disability.

It is therefore critical that researchers who are part of civil society should take a lead in generating the evidence that will be used to craft effective development programmes and policies for supporting the rights of children, women and men with disability. Without credible evidence our campaigns for an equitable world that includes both people without and people with disability as full citizens will be meaningless.

Recognising the important role of researchers, it is now the time to develop the capacity of researchers with disability. This is, in fact, what we are aiming to achieve through a new initiative that we have started with the support of the United Kingdom’s Department for International Development (DFID). The SAFOD Research Programme (SRP) is teaching us that people with disability are the best to tell their own story and to drive their own research agenda.

Through the SRP we have also learnt that collaboration in the research process is important not only for capacity building but also for effective engagement with academia and mainstream researchers. The idea is to not let people with disability continue as passengers on the research train; they must be in the engine room and steer that train.

With regard to the SRP, the criterion set by the Technical Advisory Board (TAB) that each bidder should have a person with disability as part of the research team is not merely to say people with disability should be used as window dressing. Instead, we want them to participate fully because they will soon be driving this process. To achieve this, however, we need to ensure that collaborative efforts between organisations in developed and developing countries yield mutual benefits. This must be achieved through genuinely equal (but not necessarily equivalent) partnerships; even though much of Africa does not have financial, technical and material resources, Africa does have a wealth of talented people, and this resource does and should include people with disability. Much more research on disability issues is needed for positive and effective policy development in Africa.
